# Smartwatch Sensors with Deep Learning to Predict the Purchase Intentions of Online Shoppers

**DOI:** 10.3390/s23010430

**Published:** 2022-12-30

**Authors:** Ray-I Chang, Chih-Yung Tsai, Pu Chung

**Affiliations:** 1 Department of Engineering Science and Ocean Engineering, National Taiwan University, Taipei 10617, Taiwan; 2 Department of Education, University of Taipei, Taipei 100234, Taiwan

**Keywords:** precision marketing, purchase intention, electrocardiogram, wearable device, long short-term memory

## Abstract

In the past decade, the scale of e-commerce has continued to grow. With the outbreak of the COVID-19 epidemic, brick-and-mortar businesses have been actively developing online channels where precision marketing has become the focus. This study proposed using the electrocardiography (ECG) recorded by wearable devices (e.g., smartwatches) to judge purchase intentions through deep learning. The method of this study included a long short-term memory (LSTM) model supplemented by collective decisions. The experiment was divided into two stages. The first stage aimed to find the regularity of the ECG and verify the research by repeated measurement of a small number of subjects. A total of 201 ECGs were collected for deep learning, and the results showed that the accuracy rate of predicting purchase intention was 75.5%. Then, incremental learning was adopted to carry out the second stage of the experiment. In addition to adding subjects, it also filtered five different frequency ranges. This study employed the data augmentation method and used 480 ECGs for training, and the final accuracy rate reached 82.1%. This study could encourage online marketers to cooperate with health management companies with cross-domain big data analysis to further improve the accuracy of precision marketing.

## 1. Introduction

Online stores, ad portals, and shopping apps have become part of everyday life in 2020–2021.After the outbreak of the COVID-19 pandemic, more brick-and-mortar business operators started developing their businesses through online channels [1]. Business in the online space faces new challenges that require additional knowledge and skills [2]. As compared the brick-and-mortar retail model, online marketing is more flexible and diversified. However, precision marketing is the key to the success of online marketing. In order to create stronger purchase motivations, e-commerce platforms must be able to accurately understand the behaviors and preferences of consumers. Precision marketing may help enterprises to save resources and promote consumers’ continuous shopping behaviors [3].

In terms of common precision marketing methods, consumers’ shopping behaviors, such as shopping portfolios and shopping hours, were captured via big data analysis. However, such practice was rather passive. In addition, the precision marketing implemented by e-commerce platforms uses a large amount of consumer data. As the behaviors of consumers are dynamic and diverse, it is difficult for businesses to find the products and services that consumers need from the vast amount of disordered user data [4]. Related studies suggested that electrocardiograms (ECGs) can be used to distinguish people’s emotions [5], which further help to analyze purchase intentions [6] and achieve precision marketing. Therefore, this study aims to use ECGs to directly identify consumers’ purchase intentions, which is the first main research motivation.

Wearable devices mainly collect relevant data through various sensors, including physiological data sensing, environmental data sensing, and motion sensing. Through communication interfaces, such as Bluetooth, wearable devices can be connected to smartphone apps and cloud platforms, which have a larger application space. There are many kinds of wearable devices with various functions. At present, the main technology in most smartwatches and smart wristbands is to carry out operational analyses on the collected data through a built-in accelerometer and gyroscope. Heart rate is the basic sensing data collected and is mostly used for physiological behavior analyses, such as sedentariness [7] and sleep analysis [8,9,10], which intend to promote a healthy lifestyle of the users [11]. Studies have used heart rate data to evaluate the emotional responses of the users [12,13], as well as the service experiences and advertisement stimulation of the consumers [14,15].

Wearable sensor data can also be combined with deep learning prediction, and are used further for related applications. For example, Pathinarupothi et al. [16] used wearable sensor data to predict sleep apnea (with an accuracy of 99%), Zhou et al. [17] used it to predict the learning state of students (with an accuracy of 74%), and Tsai et al. [18] used it to predict dangerous driving behavior (with an accuracy of more than 80%). Mirza et al. [19] predicted characteristics of disease infection with an accuracy of 68%, which is higher than that in the past. Other studies also predicted purchase intention by combining ECGs with in-depth learning. Therefore, this study designed a purchase intention experiment to collect the subjects’ heart rates obtained by wearable smartwatches, and further used a deep learning model to predict their purchase intentions, which is the second main research motivation.

At present, in-depth learning models are being widely applied in many fields, and good results have been obtained, such as MSISDT dataset image recognition [20], text reading comprehension [21], and electroencephalogram (EEG) wavelet transform (WT) for emotion recognition [22]. In a neural network, data volume often determines the quality of a model. Therefore, these studies applied data augmentation to produce data that are similar but not identical to training data, thus solving the data collection problem. Finally, this study intended to improve the accuracy by the sensor resampling-based augmentation method, which is the third main research motivation.

Based on the results of this study, wearable smartwatches were used to collect heart rate data, which were then classified through the lightweight model of TensorFlow Lite. Finally, commodities and their corresponding prediction results could be uploaded to the background for sellers to use.

## 2. Related Works

### 2.1. Wearable Devices

Smartwatches or smart wristbands are mainly used to measure the users’ heart and respiration rates. Heart and respiratory rate data can be used as a reference to evaluate stress, decision-making, and concentration [23,24]. The main working principle is that, when the heartbeat changes, the brain’s cortical potential also changes; this is known as the heartbeat evoked potential (HEP). Due to the synchronous recording of the heart and brain, when there is an R peak in the heart, the EEG signal shows a large negative peak at 200–600 ms simultaneously. HEP occurs when the upload paths of both the heart and brain through the vagus nerve are synchronized [25]. Starr et al. [26] studied the connection between heart rate variability (HRV) and HEP in different emotional states. They found that HRV was consistently associated with an increase in HEP amplitude, particularly during respiration with a resonant frequency. Heart rate measurements are relatively easy to take. Therefore, as long as the heart rate meter is placed on the wrist near the radial artery, the device is unlikely to interfere with the user’s activities [19]. The heart rate can also be evaluated in both a laboratory and a non-laboratory environment [27].

At present, when comparing smartwatches or smart wristbands with the standard 12-lead electrocardiography (ECG) commonly used in medical institutions, Apple claims that the ECG measured by its smartwatch is similar to a 1-lead ECG which can effectively provide information about the user’s heart rhythm, and classify arrhythmias. Apple conducted a clinical trial on about 600 subjects, recorded their ECG signals using Apple Watches, and then checked the accuracy of results classified by the App as arrhythmia or sinus rhythm. Finally, among the classifiable results, the true positive rate (TPR) of the arrhythmia classification was 98.3%, and the true negative rate (TNR) of the sinus rhythm classification was 99.6% [28].

ECG signals not only can be used as indicators to observe cardiac functions, but also analyze the users’ emotional states. Forte et al. [29] pointed out that ECG signal data can be combined with spectrum analysis to identify three indicators of sympathetic nerves and parasympathetic nerves (as shown in Table 1), and can be used to identify the emotional state of the users. Geoffrey et al. [30] measured the data from 9750 Apple Watch users from around the world, which included up to 139 million ECG measurements. Their study used heuristic pre-training to train the deep learning network. The results of their model had a sensitivity of 98% and a specificity of 90.2% for 51 patients with arrhythmia.

### 2.2. Long Short-Term Memory Applied to ECG

In deep learning, time-series data, such as ECG data, belong to the specialty of recurrent neural networks (RNNs). RNNs store the output of the hidden layer in memory. When training the next input data, the previous value stored in the memory is also considered for calculation. However, if the time series of the data is long, RNN is easily limited by short-term memory issues, causing the preceding data to become less influential and the RNN to miss important information. However, the long short-term memory (LSTM) model can be used as a solution to address the above problems. LSTM is a modified version of RNN that adds four units: the input gate, the memory cell, the output gate, and the forget gate. These units are responsible for controlling which values need to be remembered or forgotten, so as to facilitate the application of the next memory cell, increase the memory, and avoid the vanishing gradient [31].

A current study on adopting LSTM for ECG data in biometric identification reached an accuracy of 97% and above [32]. Furthermore, when the system is simplified and testing reference points are not used, the accuracy can reach over 98% [33]. Ref. [34] applied the LSTM on the prediction of cardiac disorders, and the accuracy reached 97% and above, in addition to discriminating the multiple classes present and providing a relativity of clinical perspective.

In view of the above, the combination of LSTM and ECG could provide a certain accuracy in prediction. This study used the LSTM as the in-depth learning model and accordingly designed the model and adjusted the parameters.

### 2.3. ECG Feature Extraction

The ECG data of people are often affected by the location, size, and anatomical structure of the heart. In addition, gender and the entire structure of the chest also give specificity to the ECGs, making the ECGs authentic values. The ECG signals passed through numerous filters to remove noise. Then, the Pan Tompkins algorithm was used to locate the R-peak as signal preprocessing [35].

In the study of biometric identification, Ref. [36] found the highest point of each ECG waveform, that is, the R peak as a reference point to segment, and used the length of nine ECG waveforms as input. Ref. [37] also used the band-pass filter and determined the PQRST points in each waveform. Eleven features were extracted from the results, including the amplitude attributes and timing attributes. In Refs. [32,34], ECG sequences were extracted using a 2 s rectangular window, and completed the z score standardization. The training sequences were extracted by shifting the rectangular window with an overlap of 0.75 fractions.

In view of the above, different ECG features have different effects on learning. This study applied feature augmentation and precisely identified the PQRST points that have significant effects on learning.

## 3. Research Methods

The design procedure of this study includes data collection and data augmentation; data pre-processing; model training; and testing results. This study discussed the impact of data amplification on the same model and compared it with the original data model.

### 3.1. Data Collection

Data were collected in laboratory experiments. Table 2 lists the system environment.

The experimental design of this study was divided into two stages. In the first stage, repeated experiments were carried out on the subjects. Through the collection and analysis of ECG data, regularity in the ECG data was explored to confirm the feasibility of purchase intentions. In the second stage, non-repeated experiments were carried out on the subjects. The experiments were carried out on each subject only once, in order to verify their practical application value. The smartwatches used in this experiment were Apple Watch Series 6, and each ECG measurement lasted for 30 s.

First, the subjects browsed on-line shopping pages on their laptops, while being measured by the ECG. After they understood the products and had fully thought about it, the subjects were asked whether they had purchase intention, and their answers were recorded. A total of four subjects participated in this experiment, and each subject took five to eight ECG measurements every two to three days. The subjects all viewed the same on-line shopping pages, and the data collection lasted for one month. Finally, 201 ECG data sets were collected. Among the subjects, 39% expressed purchase intention, while 61% had no purchase intention.

A key challenge in data augmentation is to accurately simulate the same type of data with information from different fields, that is, to ensure that the amplified samples have the same semantics as the original samples. At present, commonly used sensor data amplification methods include [38,39,40]. However, all these methods were migrated from the time-series augmentation method without considering the characters of sensor data or that the new samples are not good representatives of the original label data. As a result, the performance might greatly fluctuate with datasets.

**Figure 1:**
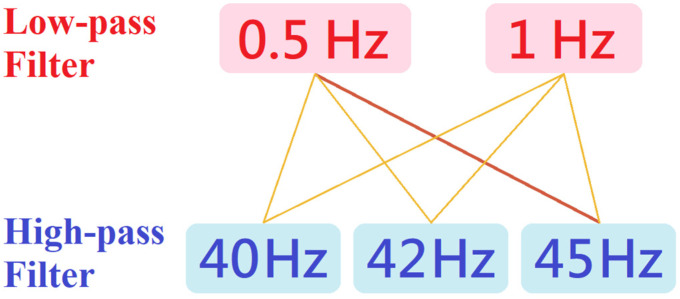
Combination of high and low frequency filters.

Based on the above, the incremental learning method was adopted for improvement. In the second stage of the experiment, the data from the first stage were included for training. In order to prevent the trained model from not being sufficiently objective or referential due to the variations in ECG data quantity from different subjects, for some subjects with few ECGs, this experiment used data amplification to unify the data of each subject, and balance the influence of each source data set on the model. Modern ECG devices offer a wide range of filtering options for signal processing. The low-frequency filter is usually set at 0.5 Hz or 1 Hz, while the high-frequency filter is set between 40 Hz and 45 Hz. Therefore, taking the adjustability of filtering into consideration, this study proposed the method of filtering out ECGs with different results to amplify the data through the combination of different high-frequency and low-frequency filters, as shown in Figure 1.

The second stage began after the researchers confirmed the experimental results of the first stage. The experimental equipment and procedures of the second stage were almost the same as those of the first stage, except that the device used to browse the on-line shopping page was changed from a laptop to a tablet, which reflected the recent trend of using mobile devices for on-line shopping. There were 12 subjects for the experiment, and 120 data sets were collected (10 measurements were taken from each subject). Each subject viewed the same on-line shopping page, and the data collection lasted for 1.5 months. In the second stage, 46% of the subjects expressed purchase intention, while 54% had no purchase intention.

### 3.2. Signal Preprocessing

**Figure 2:**
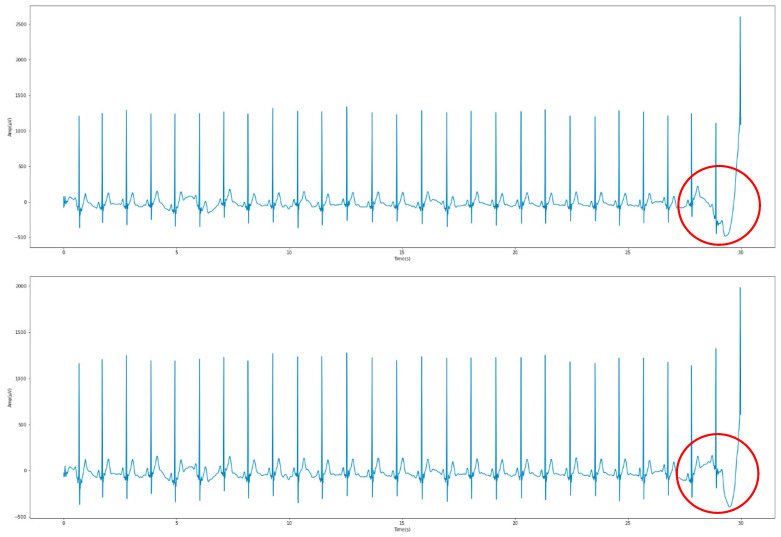
Differences before and after filtering.

After each ECG was measured, the Apple Watch transmitted the data to a connected iPhone in real-time, and each piece of data was saved as a CSV file. In order to speed up the preprocessing process, this study employed a simple Python program to format all the data in the files into a data set. Signal preprocessing in this study was divided into the four main methods of filtering, segmentation, standardization, and feature extraction. The first method involved basic filtering, which aimed to filter out the various types of noise recorded during the ECG measurement. Common noises included muscle images and baseline drift at low frequencies, as well as electromagnetic interference at high frequencies. The signals were filtered by bandpass filtering between 0.5 Hz and 45 Hz. Figure 2 shows the effects before and after the overall filtering. It could be observed that the baseline at the end of the signal was slightly adjusted.

**Figure 3:**
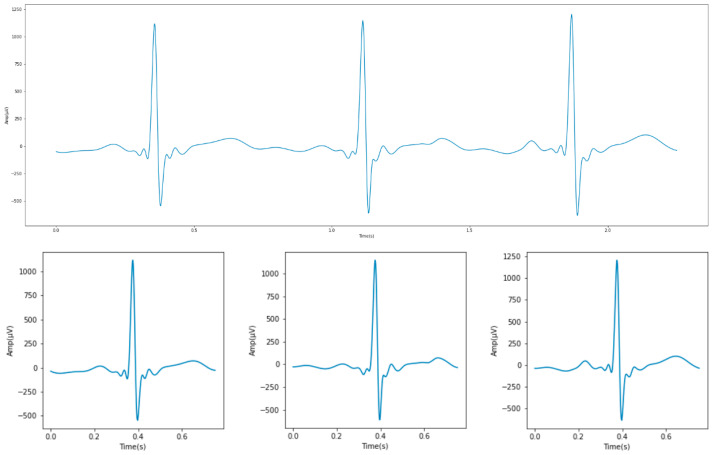
A single ECG waveform.

ECG is a kind of time series data that must be segmented before conducting deep learning prediction. The segmentation process splits and labels the data using custom time units, and then disrupts the order of the data to conduct model training. Two segmentation methods are commonly used for ECG data. One method is to use the PT algorithm to find the positions of the PQRST points of the signal so as to cut out the complete wave [35]. The other method is to use the R peak, which is the highest point of each waveform, as the reference point for segmentation [18]. The ECG measurement App of the Apple Watch S6 used in this study could fixedly sample 512 data points per second [28]. Under normal conditions, the human heart rate is 60 to 100 beats per minute. Therefore, a heart rate of 80 beats per minute was used as the average value in this experiment. Using the smartwatch for 30 s of measurement would result in about 40 heartbeats being recorded. Therefore, through the operational formula of 512 data points per second multiplied by 30 s, and then divided by 40 heartbeats, it could be estimated that there were 384 data points per heartbeat. Since the R peak is roughly in the middle of a waveform, 384/2 = 192 data points could be taken at the left and right sides of the R peak. A complete ECG waveform could be obtained by segmentation using this fixed length (Figure 3).

**Figure 4:**
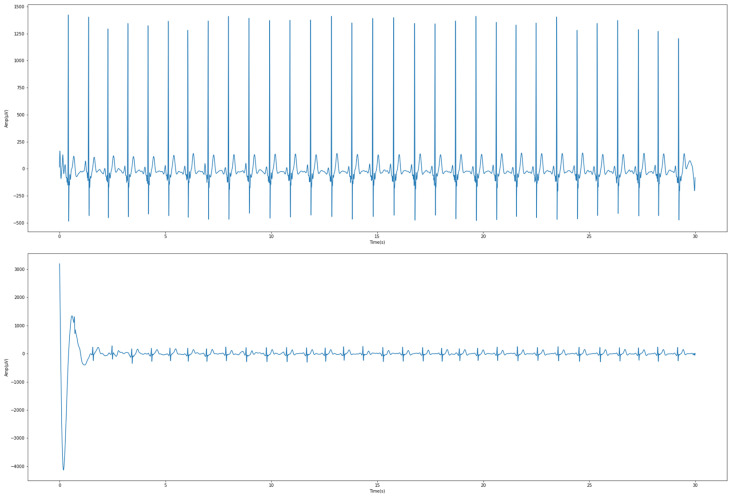
Differences in the R peaks of the ECGs for different subjects.

In general, a common ECG waveform contains a fixed band. However, the waveforms of each person are different and unique. Taking the R peak as an example, as shown in Figure 4, it was obvious that the peaks of some people are particularly high. Therefore, in order to prevent the range of the upper bounds and lower bounds of the data from being too large (which could affect the results of the model training), it was necessary to carry out standardized featurization of the data. After repeated attempts in this study, the standard range was customized between −5 and 5, so as to keep the baseline ECG at 0. In addition to being intuitive when browsing the data, this range also provided better training results compared to the relatively common standardized interval of 0 to 1.

**Figure 5:**
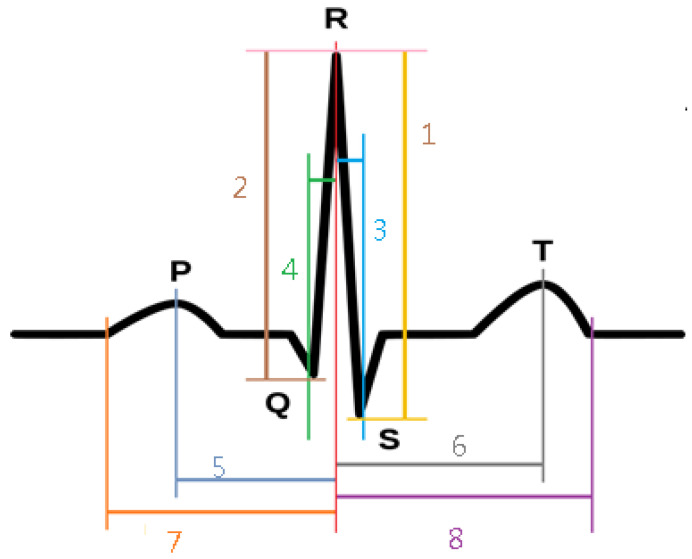
Eight ECG features and the PQRST points. (where ‘1’ is the amplitude difference between point R and point S; ‘2’ is the amplitude difference between point R and point Q; ‘3’ is time interval from point R to point S; ‘4’ is time interval from point Q to point R; ‘5’ is time interval from point P to point R; ‘6’ is time interval from point R to point T; ‘7’ is the time interval from the left of point P to point R; ‘8’ is time interval from point R to the right of point T).

After three preprocessing procedures, for the distance or height of each waveform captured by the ECG measurement App, this study added two features through the open-source NeuroKit2 suite, which is a tool specifically designed to process biological and physiological signals. The NeuroKit2 suite could find the peak values of the remaining PQRST points, but also locate the beginning and end values of each waveform [41]. A total of eight features were extracted in this study and divided into the amplitude attribute and the timing attribute, as shown in Figure 5.

### 3.3. Model Training and Calibration

**Figure 6:**
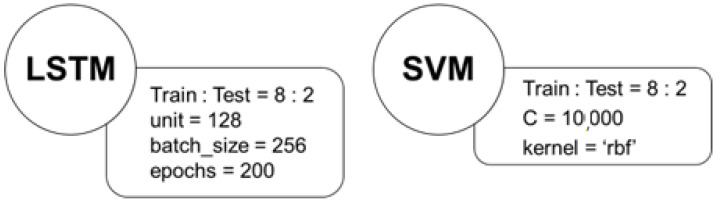
Both model parameters (where ‘rbf’ is the radial basis function).

The preprocessed data set was initially assigned a label, which was a binary classification of “0” or “1”. “0” means that the subject has no purchase intention, while “1” means that the subject has purchase intention. Then, after disrupting the order of the data, the data were divided into training sets and testing sets, at a ratio of 8:2. In this study, the ECG waveforms were first directly inputted for training through LSTM in deep learning. Then, SVM in machine learning was used as a comparison, and eight values after ECG feature extraction were input. Figure 6 shows the detailed parameters for both models.

#### 3.3.1. Long Short-Term Memory Model

**Figure 7:**
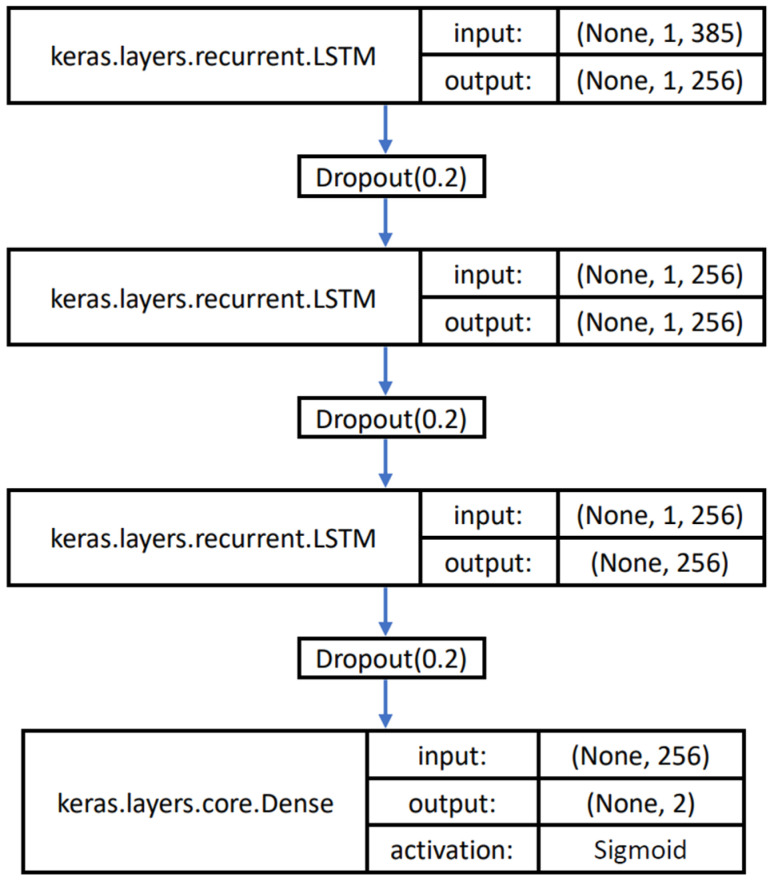
Model structure and parameters.

In this study, Keras was used to construct the three-layer model LSTM. Each layer has 256 neurons, and is connected to the fully connected layer to output the final prediction result. Figure 7 shows the structure and parameters of the model. The input factor was constructed into three dimensions. The first dimension represents the batch size of the training samples, the second dimension represents the time steps of the sequence, and the third dimension represents the units in an input sequence. In addition, the label was set based on one-hot encoding to extend the feature to a certain extent. In this study, the independent ECG waveform of Chan [42] was used as the input. In the experiment of this study, when the number of epochs was set as 200, the model could achieve a better learning rate. The nonlinear sigmoid function was selected for the excitation function. The output range of the nonlinear sigmoid function was between 0 and 1, which was in line with the prediction probability of the dichotomy result in this study.

#### 3.3.2. Support Vector Machines

Compared with the deep learning method, the supervised machine learning method, SVM, has eight input features, and its training time is faster. The penalty coefficient (C) for parameter neutralization is the weight of adjusting the classification interval size and accuracy preference. When the penalty coefficient inclines to infinity, samples with classification errors are not allowed to exist, and overfitting is prone to occur. On the contrary, when the penalty coefficient inclines to 0, a meaningful classification cannot be obtained, the calculation cannot converge, and underfitting can be found. Therefore, the penalty coefficient was set as 10,000 in this experiment.

In addition, the radial basis function (RBF), which is the most commonly used kernel function in SVM classification, was used as the kernel function. In order to transform the feature projection into a higher dimensional space, the gamma value was set as 0.5, representing the scaling ratio of the unidimensional data projected into a multidimensional space. A smaller set value would result in greater dispersion of the samples in the multidimensional space and more support vectors. The training time was prolonged, but the prediction ability was good, and it was more difficult for overfitting to occur.

#### 3.3.3. Group Decision

Group decisions can reflect collective opinions. In order to enable the model to simulate the process of group decision, this study segmented the two training data sets into five equal parts and used four non-repeated parts for training. By doing so, overfitting could be avoided due to the complexity of the model. Multiple models were used to judge and vote to improve the final accuracy.

## 4. Results

During the experimental process, the subjects were given sufficient time to browse information on the product web page before taking measurements. This ensured that all ECG waveforms from the same measurement belonged to one label. In the first stage, 201 ECGs were collected and measured. After filtering and segmentation, there were a total of 6955 single ECG wave patterns. Then, the values were uniformly standardized between −5 to 5, and eight additional features were extracted for SVM.

Table 3 shows the difference between the SVM results and the LSTM results based on deep learning, so as to understand the improvement of the vanishing gradient through LSTM and show its advantages for time series data. In addition, when the two methods were combined with group decisions, the accuracy was further improved by 1–3%. Moreover, in terms of the training and test data and the accuracy rate, the model had no obvious overfitting.

The first stage of the experiment aimed to find the regularity in ECGs through repeated measurements from a small group of subjects. Moreover, the feasibility of this study could be confirmed according to the accuracy of the model results. Therefore, in order to verify the practical application of the proposed method, the experiment in the second stage collected and measured a wider range of different ECGs from multiple non-repeated subjects.

In the second stage of the experiment, 120 ECGs were collected and measured from 12 non-repeated subjects. In addition, five different frequency ranges were used for filtering, and the ECG data of each subject were augmented to a uniform quality. This method ensured that data sets from different sources would have an even influence on the model. The other signal preprocessing steps were the same as the model training parameters in the first stage. The final research results are shown in Table 4, which lists the change trend of the data using four subjects, eight subjects, and 12 subjects. The accuracy rate reached more than 80% and had a slight increase.

## 5. Discussion

Previous studies pointed out that ECGs can be used as an important reference for judging emotion, and that emotion is highly correlated with purchase intention. This study designed a two-stage experiment to measure the subjects’ purchase intention. It collected ECG recordings from Apple Watches, and 512 data points were sampled per second. The prediction and voting system, based on the complete ECG waveform and the LSTM model with 192 data points respectively on the left and right sides of the R peak, had an accuracy of 75.5%. Compared with the 70.6% accuracy of the prediction and voting system of the SVM model, as well as [18,43] the PT algorithm used to find the positions of the PQRST points for the signal, this result was more accurate.

When a large amount of data enters machine learning, the learning parameters from the old data would be adjusted after each learning from the new data. With continuous updating and adjustment, the learning parameters of the earliest data would be completely covered or forgotten. This common defect is referred to as catastrophic forgetting. The simplest and most intuitive solution for catastrophic forgetting is to retrain all known data sets; however, this method is inefficient and cannot learn new data in a timely manner. The main goal of incremental learning is to find the most effective balance point between model stability and plasticity using limited computing and storage resources. The advantage of incremental learning is that it can constantly process newly inputted data. In addition to constantly updating the model parameters, incremental learning can also retain better parameter results in the original model.

In this study, incremental learning was adopted to carry out the second stage of the experiment. In addition to adding subjects, it also filtered five different frequency ranges. Through these different filtering ranges, the data sets of each subject could be amplified to a uniform quality, so as to ensure that different subjects would have the same weight of influence on the model. Then, the change trend based on four subjects, eight subjects, and 12 subjects was observed, and the accuracy was 81.5%, 81.9%, and 82.1%, respectively. This result also showed that the incremental learning process was stable and had a practical effect on the accuracy of this study.

The results of this study could serve as a reference for e-commerce platforms. When consumers browse product web pages, real-time ECG data can be measured by a smartwatch, transmitted to a smartphone by Bluetooth, and then uploaded to a cloud system. Through the corresponding member identification or product name, consumers can then receive recommendations to purchase products. The websites can directly and explicitly use target products to advertise to consumers, without the need for random marketing based on the user’s browsing history. Precision marketing can thus improve sales performance.

## 6. Conclusions

This study designed a purchase intention experiment in which ECG data were collected by smartwatches and purchase intention was predicted by the LSTM model, along with collective decisions. The experiment was divided into two stages. The first stage of the experiment determined the regularity of ECG and verified the feasibility of this study through repeated measurements of a small number of subjects. A total of 201 ECGs were collected for deep learning, and the results showed that the accuracy of the purchase intention prediction was 75.5%. The second stage included multiple non-repeated subjects and used the data augmentation method. In the second stage, 480 ECGs were used for training, and the final accuracy rate reached 82.1%.

According to the results, this study identified the features that are significantly influenced by ECG signals, applied the feature augmentation technique, and enlarged ECG datasets for in-depth learning. The prediction result shows that the proposed method can improve prediction accuracy, and directly recognize consumer purchase intentions by combining ECG and in-depth learning or machine learning. The findings of this study can provide a reference to e-commerce platforms in developing Apps. When consumers wear smartwatches equipped with the function for taking ECG measurements, the smartwatches could measure the ECG data in the background and transmit the data to the consumers’ smartphones via Bluetooth. When consumers browse product websites, the App could obtain real-time EGCs for data pre-processing and model classification. Finally, the model classification results could be uploaded to the backend (cloud). Advertisements of target products can be displayed precisely to consumers according to the ECG recognition results, thereby eliminating the need for random marketing based on browsing history. In addition, from the perspective of individual commodities, precise marketing plans can be devised based on the marketing performance and potential of different consumer groups and shopping hours.

## Figures and Tables

**Table 1:** HRV parameter table.

Index	Clinical Significance
Standard Deviation of Normal to Normal R Wave (SDNN)	Global HRV
Root Mean Square of the Successive Differences (r-MSSD)	Related to parasympathetic nerve activity
Number of pairs of successive NNs that differ by more than 50 ms (NN50)	

**Table 2:** Experiment execution environment.

Operating System	Program Version	CPU	GPU
Windows 10	Python 3.8 Keras 2.6.0	AMD R5 3600	RTX 2060

**Table 3:** Comparison of the experimental results in the first stage.

Question	Purchase Intent Prediction—1st Experiment	
Data Sources	ECG measured by Apple Watch Series 6	
Data Filtering	0.5~45 Hz bandpass filter	
Data Segmentation	R point left and right fixed length cutting	
Number of Waveforms	6955	
Standardization	−5~5	
Feature Extraction	None	Extract 8 features
Group Decision	5 model results to vote	
Model Input	single ECG waveform	8 features
Model	LSTM	SVM
Training Set Accuracy	79.4%	76.0%
Test Set Accuracy	72.4%	69.5%
Group Decision Accuracy	75.5%	70.6%

**Table 4:** Comparison of the experimental results in the second stage.

Question	Purchase Intent Prediction—2nd Experiment		
Data Sources	ECG measured by Apple Watch Series 6		
Data Filtering	5 filter ranges to achieve data amplification		
Data Segmentation	R point left and right fixed length cutting		
Standardization	−5~5		
Group Decision	5 model results to vote		
Model Input	single ECG waveform		
Model	LSTM		
Number of people	4	8	12
Training Set Accuracy	88.3%	87.5%	86.2%
Test Set Accuracy	80.0%	80.1%	81.8%
Group Decision Accuracy	81.5%	81.9%	82.1%

## Data Availability

Data available on request due to privacy.
